# Immunotargeting of tumor vasculature: preclinical development of novel antibody-based imaging and therapy against TEM1/CD248

**DOI:** 10.1186/2051-1426-1-S1-P81

**Published:** 2013-11-07

**Authors:** Chunsheng Li, Junying Wang, Jia Hu, Ann Marie Chacko, Aizhi Zhao, Chaitanya Divgi, Vladimir Muzykantov, George Coukos

**Affiliations:** 1 Department of Obstetrics and Gynecology, University of Pennsylvania, Philadelphia, PA, USA; 2 Department of Radiology, University of Pennsylvania, Philadelphia, PA, USA; 3 Department of Pharmacology, University of Pennsylvania, Philadelphia, PA, USA; 4 Department of Radiology, Columbia University, New York, NY, USA

## 

The success of antibody-based theranostics depends on the identification of tumor specific biomarkers and the development of corresponding antibodies with high-affinity and specificity. Tumor endothelial marker-1 (TEM1) is highly expressed in tumor vasculature of multiple cancers but not in normal organs. The expression of TEM1 was first evaluated and confirmed by immunohistochemistry from 53 cases of metastatic serous ovarian cancer at HUP. TEM1 positive tumor stroma was observed in >95% of the cases studied. Hence, developing sensitive and effective theranostic agents against TEM1 are of utmost significance in improving diagnosis and treatment of ovarian cancer. Our goals are: 1) engineer TEM1-specific antibodies; 2) evaluate these engineered antibodies in imaging and immunotherapies in preclinical models. To generate TEM1-targeting agents, we designed a panel of multivalent fusion proteins from scFv78, a previously isolated single chain variable fragment specifically recognizing the extracellular domain of TEM1. scFv78 was fused with different huIgG1 Fc region (CH2-, CH3-, or hinge). Proteins were expressed in 293F cells and purified by affinity chromatography. All scFv78 variants exhibited comparable thermo and serum stability in vitro. Among them, the scFv78-Fc fusion (78Fc) has the highest affinity to TEM1 (Kd = 0.15nM, 15X higher than scFv78). Pharmacokinetics (PK) and biodistribution of the protein panel were evaluated in naïve and TEM1+ tumor bearing animals. 78Fc has a t1/2 of 5.1hr, which is suitable for in vivo therapeutic and imaging applications. Therefore, 78Fc was further developed as imaging tool and antibody-drug conjugate (ADC) based on its favorable affinity, stability, half-life and PK profile. In pilot studies with preclinical animal models of tumor vasculature, fluorophore- and [124-I]-labeled 78Fc demonstrated specific enrichment in TEM1+ grafts, but not in control tumor or other organs, by both optical and immunoPET imaging. In addition, 78Fc-MMAE conjugate exerted specific killing of TEM1+ cells. In summary, we have developed a panel of innovative theranostics agents targeting TEM1 on the vasculature of ovarian cancer and several other solid tumors. Our long term goal is to translate such combined approach into the clinic: Using TEM1-antibody as imaging tools to select, and monitor patients for TEM1-antibody based targeted therapies.

